# Short-term effects of distant-image screen and myopic defocus on choroidal thickness in children: a randomized pilot clinical trial

**DOI:** 10.3389/fpubh.2026.1884932

**Published:** 2026-09-04

**Authors:** Xingyi Guo, Chuanli Zhang, Yiyuan Wu, Zhanliang Ruan, Wenli Lu, Xiaoqin Chen, Lihua Li

**Affiliations:** 1 Tianjin Eye Hospital, Tianjin Key Lab of Ophthalmology and Visual Science, Tianjin, China; 2 Tianjin Eye Hospital Optometric Center, Tianjin, China; 3 Nankai University Optometry & Vision Science Institute, Tianjin, China; 4 Department of Epidemiology and Health Statistics, Tianjin Medical University, Tianjin, China

**Keywords:** choroid thickness, DIMS, distant-image screen, myopia, near work

## Abstract

**Purpose:**

This study aimed to characterize the changes in choroidal thickness (ChT) in response to optical approaches using the distant-image screen (DIS) device and defocus-incorporated multiple segments (DIMS) lenses.

**Design:**

This is a prospective, randomized, controlled, crossover trial.

**Participants:**

A total of 40 children (8–13 years of age) with spherical equivalent refraction (SER) ranging from −0.50 to −6.00 diopters (D) were recruited and randomized into 4 groups: children who watched videos using either a DIS or a tablet computer (TC) and those wearing DIMS or single-vision lenses (SVL).

**Methods:**

In their assigned groups, children watched videos for 90 min in stage I, followed by a 3–7-day recovery period. After this recovery period, the children in the TC group were crossed over to the DIS group, and those in the DIS group were crossed over to the TC group for an additional 90 min in stage II. The SER was measured before and after watching videos using an open-field autorefractor. The ChT in the central (1 × 1 mm), superior, inferior, nasal, and temporal regions in 1–3 mm (S3, I3, N3, and T3) and 3–6 mm (S6, I6, N6, and T6) were measured before and after watching videos using full-range swept-source optical coherence tomography (SS-OCT).

**Main outcome measures:**

The main outcome measure was the changes in regional ChT.

**Results:**

After 90 min of video watching, the SER in the TC group significantly increased after viewing 90-min videos (−2.14 ± 1.15 D vs.-2.26 ± 1.16 D, *p* = 0.036), whereas no significant difference was observed in the DIS group. Among the participants wearing SVL, the ChT changes in the DIS group were significantly higher than those in the TC group at I3 (*p* = 0.036) and T3 (*p* = 0.032), and the changes in other regions were not significantly different between the two groups. For participants using TC, the DIMS group showed greater ChT thickening at C (9.54 ± 3.19 μm, *p* = 0.005), I3 (6.44 ± 3.04 μm, *p* = 0.043), N3 (9.10 ± 3.17 μm, *p* = 0.007), and T3 (8.00 ± 3.17 μm, *p* = 0.017) than the SVL group. The combination of DIMS and DIS exhibited greater ChT thickening at I3 (11.23 ± 4.07 μm, *p* = 0.009) compared to the SVL and TC groups.

**Conclusion:**

Short-term long-distance working mode and myopic defocus may exert beneficial effects on choroidal variation in myopic children.

**Clinical Trial Registration Number:**

https://www.chictr.org.cn/showproj.html?proj=184978, Identifier ChiCTR2200065741.

## Introduction

Myopia has emerged as a major public health issue (1). The rising prevalence in myopia and younger age of onset frequently increase the risk of high myopia (2), which not only causes the disease burden of ocular pathologies such as glaucoma, cataract, and retinal detachment but also places an economic burden on families and the healthcare system (3). Thus, it is necessary to explore management strategies that can slow the progression of myopia to minimize the likelihood of associated complications later in life.

Near-work activities are considered a major environmental risk factor for the onset and development of myopia (4), since increasing educational pressures, prolonged near work, and close working distances have all been found to be associated with myopia progression (5–7). To reduce the time children spend on reading and other near work, the distant-image screen (DIS) used in this study can convert near real images into virtual images of more than 5 meters, achieved using a birdbath optical solution and free-form surface technique. Zhen et al. have shown that children using DIS avoided the decline in visual acuity caused by near viewing compared with printed matter (8), and the potential roles of DIS in myopia control require further investigation.

Defocus-incorporated multiple segments (DIMS) spectacle lenses, as a myopic defocus intervention, were shown to slow myopia progression among school-aged children in a 2-year trial (9). Choroid has been claimed to be of importance during ocular growth and refractive error development (10). In humans, short-term exposure to myopic and hyperopic defocus has also elicited a transient increase or decrease in choroidal thickness (ChT), respectively (11–14). Current clinical methods, i.e., atropine (15), orthokeratology (16) and multifocal gas permeable contact lenses (17), also reportedly produced a ChT increase. A DIMS study demonstrated that choroidal thickening occurred after 1 week of wearing the lenses and persisted over the 2-year study period (18). However, few studies have focused on the short-term changes in the choroid in response to DIMS lenses within hours.

In this crossover study, we aim to provide a better understanding of regional ChT changes in response to DIS or the combination of DIS and DIMS in children using full-range swept-source optical coherence tomography (SS-OCT). DIS is expected to provide novel strategies for myopia control treatments.

## Methods

### Participants

A total of 40 Chinese children from the Tianjin Eye Hospital Optometric Center were recruited for this study between January 2023 and June 2023. This study was approved by the Ethics Committee of Tianjin Eye Hospital (KY202223) and registered in the Chinese Clinical Trial Registry (registration number: ChiCTR2200065741). This study conforms to the tenets of the Declaration of Helsinki. All participants and their parents or guardians provided verbal and written informed consent before the procedure.

The inclusion criteria were as follows: (1) age 8–13 years; (2) spherical equivalent refraction (SER) of −0.50 diopter (D) to −6.00D in both eyes; (3) astigmatism of less than 1.50 D; (4) anisometropia of less than 1.50 D; (5) corrected distance visual acuity (CDVA) of less than 0.0 LogMAR. The exclusion criteria were as follows: abnormal accommodative function, ocular organic diseases, abnormal eye alignment or eye movement disorders, and the inability to comply with the study visit schedule.

### Distant-image screen (DIS)

**Figure 1:**
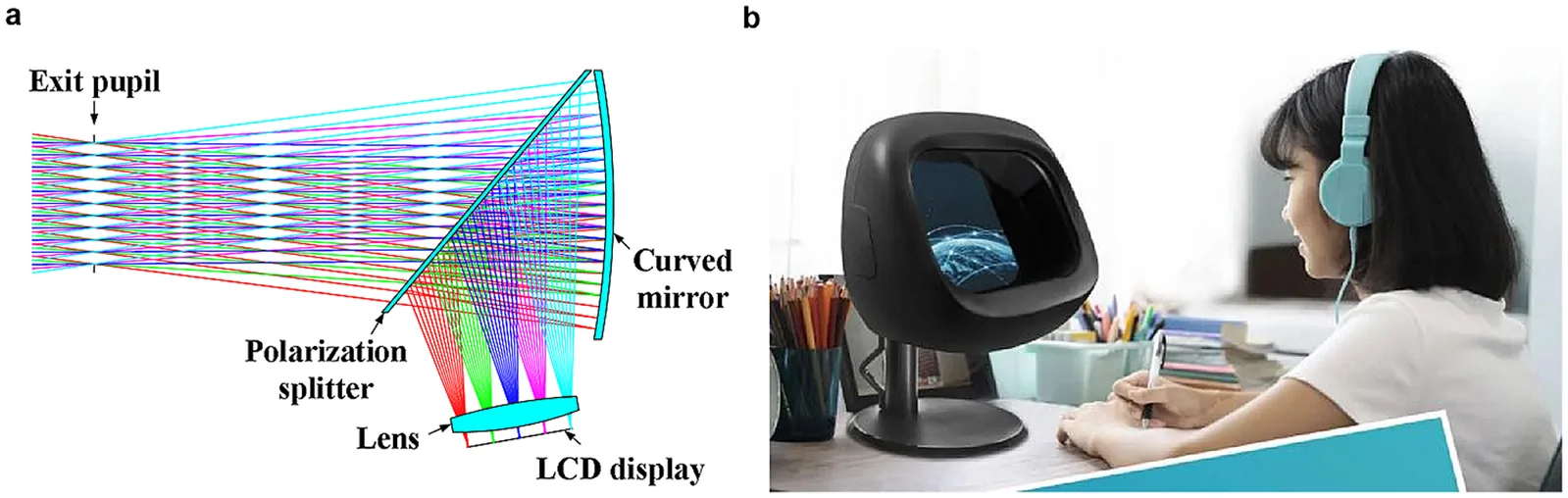
**(a)** Optical layout of the distant-image system. **(b)** Prototype of the distant-image screen.

To generate a virtual image far away from the human eye, a birdbath optical solution was used, as shown in Figure 1a. This optical system included a planar split element and a curved mirror. The lens is not necessary if the field curve of the virtual image is acceptable. Through the two/three optical elements, the image from the LCD display is projected over the real-world view. The resolution of the LCD display should be at least 1920 × 1,080. The distance between the virtual image and the human eye should be longer than 5 meters. To guarantee the optical efficiency of the system, a polarization splitter (PS) film, a quarter-wave plate, and a polarizer were used. A PS is bonded to the plate glass, the quarter-wave plate is then bonded to the PS, and the polarizer is bonded to the LCD display. In the birdbath solution, although the optical axis direction is rotated several times, the lens remains coaxial in the optical system, thereby significantly reducing the aberrations introduced by the off-axis structure. The birdbath solution can achieve a field of view (FOV) greater than 30° and an image distortion of less than 1.5%. Figure 1b shows the prototype of the DIS.

### Study design

**Figure 2:**
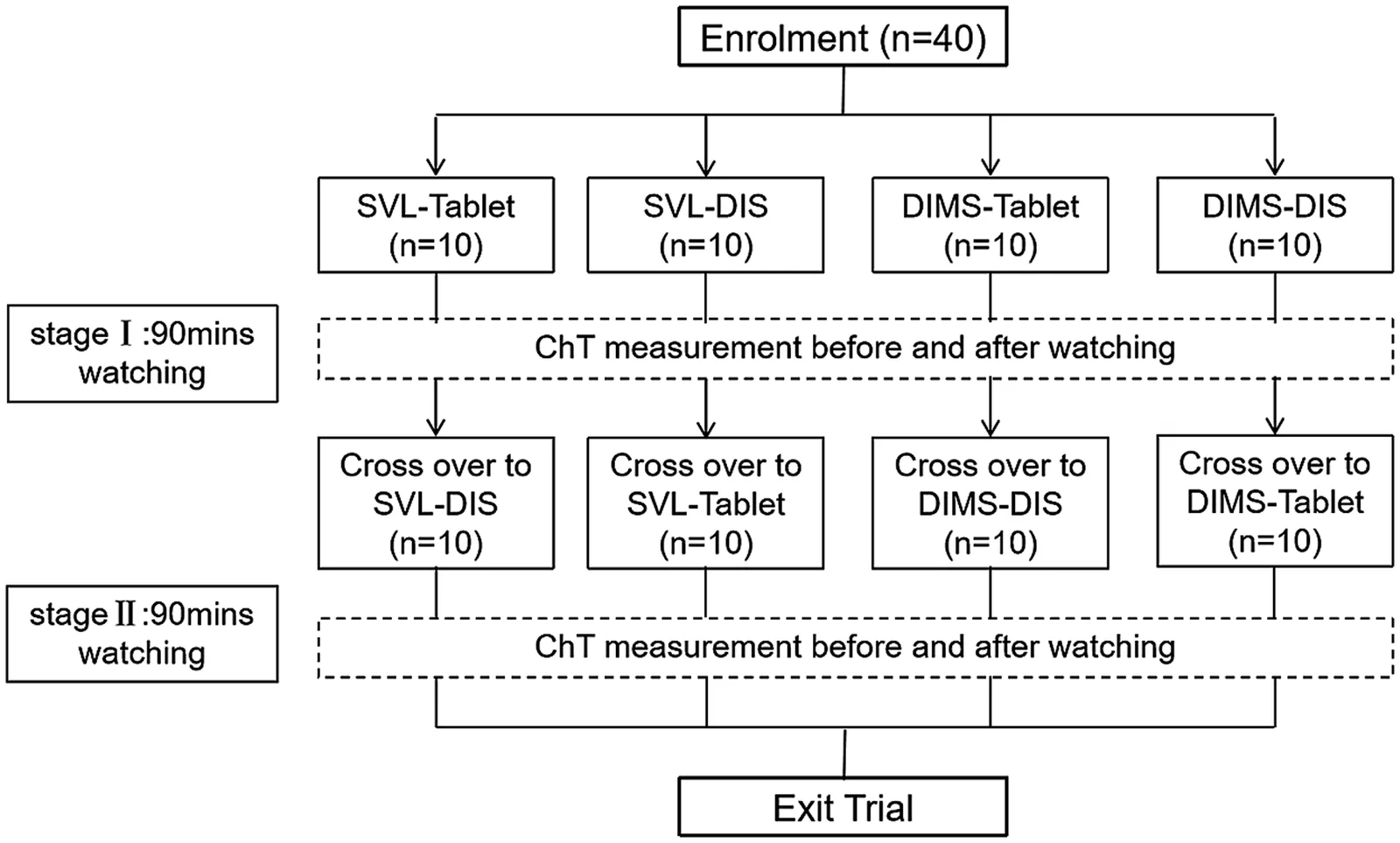
Flow of participants in the trial.

The enrolled children were recruited and randomized into four groups: wearing DIMS (Hoya, Japan) or single-vision lenses (SVL) and watching videos using either a DIS or a tablet computer (TC) (Figure 2). In their assigned group, children watched videos for 90 min in stage I, followed by a 3–7-day recovery period. Then, the children in the tablet group were crossed over to the distant-image screen group, and those in the distant-image screen group were crossed over to the tablet group for an additional 90 min in stage II. A block randomization sequence of block size 4 was generated by SAS 9.2 software (SAS Institute Inc., Cary, NC, USA) (Supplementary Table 1).

### Research procedures

Before the experiment, each participant underwent basic examinations, including uncorrected distance visual acuity (UDVA), corrected distance visual acuity (CDVA), and spherical equivalent error (SER). After basic examinations, a full correction (DIMS or SVL) was prescribed for each participant. To eliminate the effects of any visual tasks the participants may have performed before the visit, they were required to rest in a dark room for 5 min. This was followed by a viewing activity, which involved watching a video on the DIS or an Android tablet computer (HONOR AGM3-W09HN, 10.1-inches, 1920 × 1,200 resolution, 300-nit luminance) at a distance of 30 cm for 90 min. The room’s lighting was maintained at 300 lx.

The participants watched videos of interest to them under the supervision of an investigator to ensure that they were not distracted. The investigator recorded the video number and checked the viewing distance every 5 min. In each visit, the participants were asked to summarize what they had watched to ensure they were actively watching. Before and after the completion of the viewing task, the continuous 30-s dynamic SER was measured at a distance using a Grand Seiko WAM-5500 open-field infrared autorefractor (Grand Seiko Co., Ltd., Hiroshima, Japan), yielding the mean value of SER. Meanwhile, the participants moved to the SS-OCT device (YG-100 K PRO, TowardPi Medical Technology, China), and choroidal images were captured before and after watching.

### Measurement of ChT

**Figure 3:**
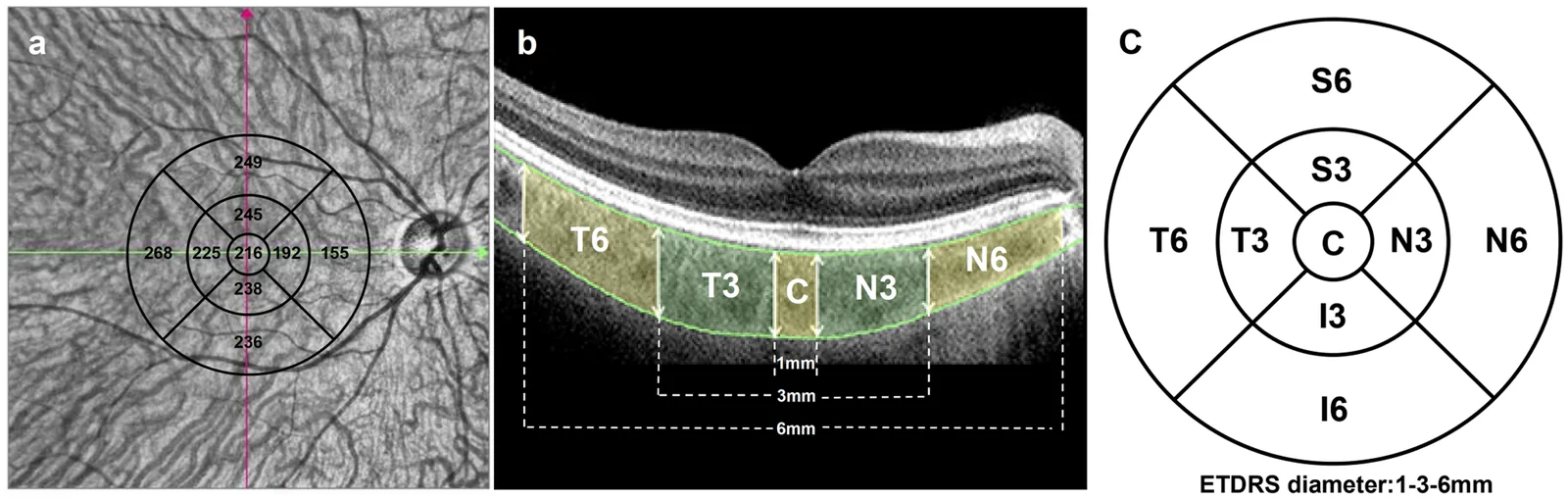
Regions of the choroid are shown in the image **(a)** and an example of a typical B-scan centered on the fovea **(b)**, and a schematic diagram **(c)**. C, 1 × 1 mm circular region; S3, I3, N3, and T3, the superior, inferior, nasal, and temporal regions measured at 1–3 mm; S6, I6, N6, and T6, the superior, inferior, nasal, and temporal regions measured at 3–6 mm.

The examinations were conducted simultaneously to minimize the influence of diurnal rhythms. The SS-OCTA system features a swept-source laser at 1060 nm and a remarkable tracking speed of 100,000 sweeps per second. The axial resolution, lateral resolution, and posterior scan depth were 3.8 μm, 10 μm, and 6 mm, respectively. OCTA fundus images were obtained three times with a raster scan protocol of 512 horizontal B-scans that covered an area of 12 mm × 12 mm centered on the fovea (Figure 3a). The ChT was defined as the thickness between the outer retinal pigment epithelium and the inner choroidoscleral interface, which was automatically detected by the machine-built algorithm and manually corrected if necessary (Figure 3b). A central circular region measuring 6 × 6 mm was automatically partitioned according to the Early Treatment Diabetic Retinopathy Study (ETDRS). The nine regions were classified as follows (Figure 3c): the central fovea, consisting of a 1 × 1 mm circular region (C); and superior, inferior, nasal, and temporal regions measured at 1–3 mm (S3, I3, N3, and T3) and 3–6 mm (S6, I6, N6, and T6). The built-in software automatically provided the thickness in each direction and calculated the average thickness of three images in each area. The results of the three measurements obtained by the two observers were averaged and used for data analysis. Changes in ChT in stage I were calculated as the differences between the pre- and post-viewing assignments; also, changes in stage II were calculated as the differences between the post-viewing and the stage II baseline.

### Statistical analysis

The per-protocol dataset was used for data analysis. SPSS 25.0 was used to analyze the data. Only right-eye data were used for statistical analysis. The Shapiro–Wilk test was used to assess normality. The mean value ± standard deviation was used to describe continuous variables. Paired or independent t-tests or paired rank-sum tests were used to compare groups depending on whether the data were normally distributed. The differences in ChT were compared using a two-stage cross-design ANOVA, with a two-sided test at *α* = 0.05.

## Results

A total of 40 child participants completed the study, including 22 males and 18 females, with an average age of 10.58 ± 1.48 years. The trial flowchart is shown in Figure 2. At baseline, no differences were found in SER, UDVA, CDVA, and ChT (nine regions) among the four groups (All *p* > 0.05; Table 1). Additionally, all participants recovered to baseline ChT after the 3–7-day recovery period (Supplementary Table 2).

**Table 1:** Baseline parameters of the participants in the four groups (mean ± SD).

Variable	SVL-TC (*n* = 10)	SVL-DIS (*n* = 10)	DIMS-TC (*n* = 10)	DIMS-DIS (*n* = 10)
SER (D)	−2.17 ± 1.29	−2.18 ± 1.22	−2.55 ± 1.22	−2.01 ± 1.05
UDVA	0.28 ± 0.10	0.30 ± 0.16	0.32 ± 0.13	0.31 ± 0.15
CDVA	1.06 ± 0.13	1.10 ± 0.11	1.06 ± 0.13	1.06 ± 0.13
C (μm)	291.70 ± 64.58	274.89 ± 54.15	263.50 ± 51.15	257.40 ± 48.88
S3 (μm)	285.60 ± 52.46	255.60 ± 66.71	265.70 ± 52.48	258.40 ± 54.31
I3 (μm)	284.80 ± 74.17	268.30 ± 75.18	270.40 ± 52.56	263.80 ± 47.74
N3 (μm)	259.70 ± 63.56	222.20 ± 68.98	236.70 ± 57.86	225.50 ± 46.39
T3 (μm)	295.50 ± 56.09	276.70 ± 62.54	273.70 ± 53.75	267.30 ± 46.71
S6 (μm)	285.70 ± 60.48	265.50 ± 44.94	268.50 ± 59.59	260.20 ± 50.12
I6 (μm)	278.20 ± 76.72	262.00 ± 81.54	268.40 ± 52.93	259.20 ± 36.84
N6 (μm)	205.00 ± 56.85	188.20 ± 49.17	188.50 ± 55.64	180.50 ± 40.38
T6 (μm)	291.70 ± 50.07	280.10 ± 45.23	274.40 ± 56.47	275.90 ± 46.93

SVL-TC, wearing SVL and watching TC; SVL-DIS, wearing SVL and watching DIS; DIMS-TC, wearing DIMS and watching TC; DIMS-DIS, wearing DIMS and watching DIS; C, 1 × 1 mm circular region; S3, I3, N3, and T3, the superior, inferior, nasal, and temporal regions measured at 1–3 mm; S6, I6, N6, and T6, the superior, inferior, nasal, and temporal regions measured at 3–6 mm; SVL, single-vision lenses; DIMS, defocus incorporated multiple segments lenses; TC, tablet computer; DIS, distance-image screen; SER, spherical equivalent error; UDVA, uncorrected distance visual acuity; CDVA, corrected distance visual acuity.

Among participants wearing SVL, the SER in the TC group increased significantly after viewing 90-min videos (−2.14 ± 1.15 D vs.-2.26 ± 1.16 D, *p* = 0.036, Table 2), whereas no significant difference in SER values was observed in the DIS group after video viewing. Additionally, the SER in the TC group was higher than that in the DIS group after watching (−2.26 ± 1.16 D vs. −2.18 ± 1.12 D, *p* = 0.036). Among participants wearing DIMS, we did not witness the above significant changes in the TC and DIS groups.

**Table 2:** SER before and after reading in the four groups (mean ± SD).

Variable	Group	SVL			DIMS		
		Before	After	P1	Before	After	P1
SER (D)	TC	−2.14 ± 1.15	−2.26 ± 1.16	0.036*	−2.30 ± 1.17	−2.34 ± 1.29	0.315
	DIS	−2.21 ± 1.23	−2.18 ± 1.12	0.501	−2.37 ± 1.17	−2.41 ± 1.19	0.247
	P2	0.825	0.036*		0.329	0.942	

SVL, single-vision lenses; DIMS, defocus incorporated multiple segments lenses; TC, tablet computer; DIS, distance-image screen.

P1: Paired t-tests were used to analyze the differences in SER before and after reading in the DIS or TC group.

P2: Independent t-tests were used to analyze the differences in SER between the DIS and TC group.

^*^Significant difference.

**Figure 4:**
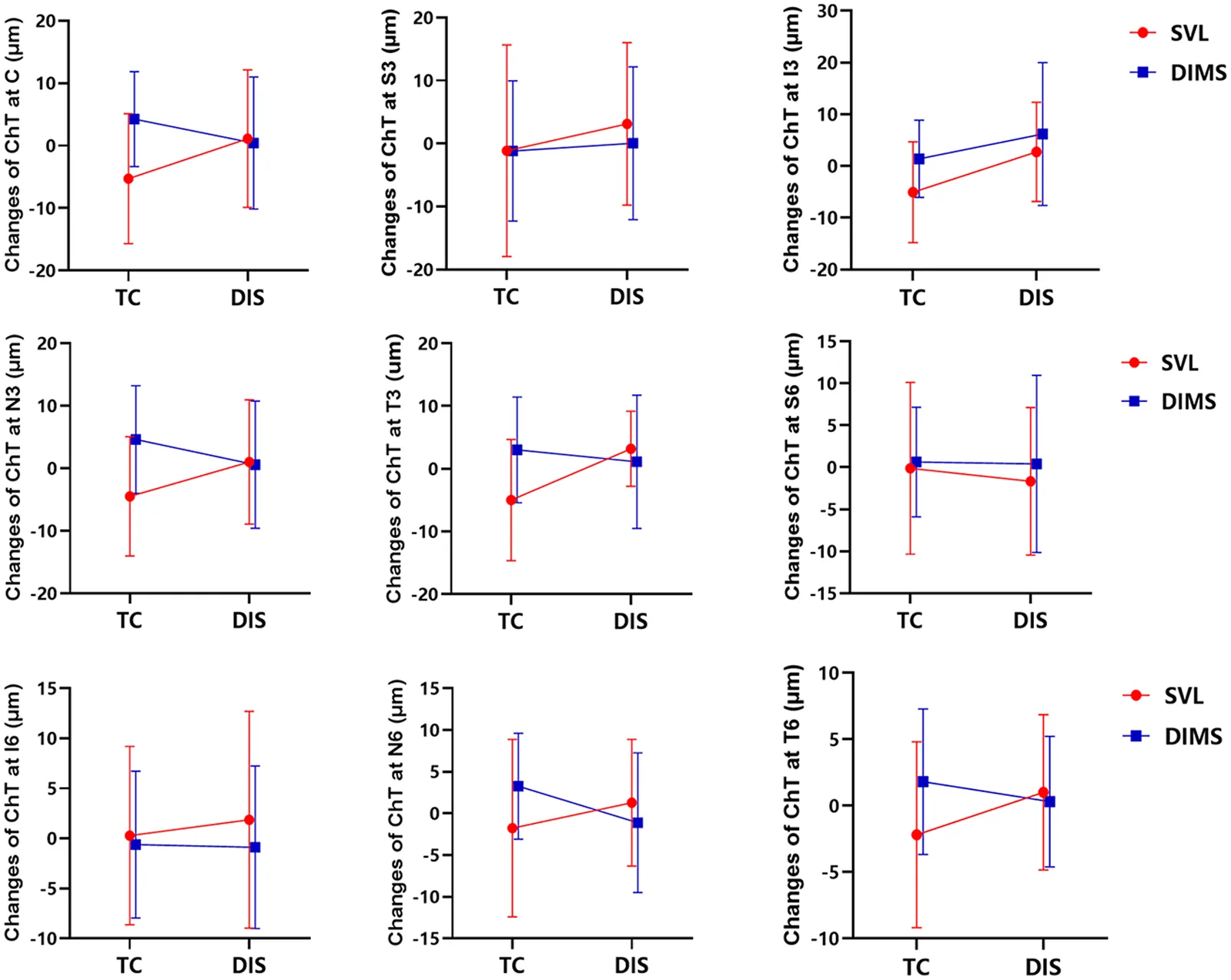
Average (and SD bars) of the changes of ChT in both stages between different groups for the nine choroidal regions. C, 1 × 1 mm circular region; S3, I3, N3, and T3, the superior, inferior, nasal and temporal regions measured at 1–3 mm; S6, I6, N6, and T6, the superior, inferior, nasal, and temporal regions measured at 3–6 mm. SVL, single-vision lenses; DIMS, defocus incorporated multiple segments lenses; TC, tablet computer; DIS, distance-image screen.

After 90 min of video watching (Table 3), a paired t-test revealed that among participants wearing SVL, ChT changes at I3 (mean difference of 7.77 ± 11.69 μm, *p* = 0.032) and T3 (mean difference of 8.18 ± 9.69 μm, *p* = 0.026) in the DIS group were greater compared to the TC group, and a similar tendency can be observed in other regions except S6 in Figure 4. For participants using TC, the DIMS group showed a higher degree of choroidal thickening at C (mean difference of 9.54 ± 3.19 μm, unpaired t-test, *p* = 0.005), I3 (mean difference of 6.44 ± 3.04 μm, *p* = 0.043), N3 (mean difference of 9.10 ± 3.17 μm, *p* = 0.007), and T3 (mean difference of 8.00 ± 3.17 μm, *p* = 0.017) compared to the SVL group. The DIMS + DIS group exhibited greater ChT thickening at I3 (mean difference of 11.23 ± 4.07 μm, *p* = 0.009) compared with the SVL + TC group. No significant differences were found between participants wearing DIMS (DIS group vs. TC group) and participants watching DIS (DIMS group vs. SVL group).

**Table 3:** Changes in both stages choroidal thickness in the four groups (mean ± SD).

Regions	SVL		DIMS		P1	P2	P3	P4	P5
	TC	DIS	TC	DIS					
C (μm)	−5.29 ± 10.41	1.12 ± 11.01	4.25 ± 7.59	0.41 ± 10.56	0.127	0.553	0.005^*^	0.850	0.412
S3 (μm)	−1.76 ± 16.79	3.11 ± 12.89	−1.18 ± 11.14	0.06 ± 12.14	0.373	0.725	0.905	0.477	0.719
I3 (μm)	−5.06 ± 9.76	2.71 ± 9.60	1.38 ± 7.48	6.17 ± 13.82	0.032^*^	0.130	0.043^*^	0.399	0.009^*^
N3 (μm)	−4.47 ± 9.56	1.00 ± 9.95	4.63 ± 8.61	0.59 ± 10.16	0.164	0.358	0.007^*^	0.906	0.145
T3 (μm)	−5.00 ± 9.69	3.19 ± 6.00	3.00 ± 8.45	1.12 ± 10.65	0.026^*^	0.985	0.017^*^	0.500	0.089
S6 (μm)	−0.12 ± 10.23	−1.67 ± 8.78	0.63 ± 6.54	−1.53 ± 9.00	0.497	0.438	0.807	0.964	0.672
I6 (μm)	0.29 ± 8.93	1.87 ± 10.84	−0.63 ± 7.33	−0.89 ± 8.12	0.688	0.241	0.750	0.411	0.685
N6 (μm)	−1.76 ± 10.65	1.29 ± 7.59	3.25 ± 6.37	−1.11 ± 8.38	0.838	0.136	0.114	0.381	0.842
T6 (μm)	−2.24 ± 7.00	1.00 ± 5.86	1.81 ± 5.49	0.31 ± 4.92	0.385	0.812	0.075	0.725	0.234

C, 1 × 1 mm circular region; S3, I3, N3, and T3, the superior, inferior, nasal, and temporal regions measured at 1–3 mm; S6, I6, N6, and T6, the superior, inferior, nasal, and temporal regions measured at 3–6 mm; SVL, single-vision lenses; DIMS, defocus incorporated multiple segments lenses; TC, tablet computer; DIS, distance-image screen.

P1: Paired t-tests were used to analyze the differences between the DIS and TC groups when wearing SVL.

P2: Paired t-tests were used to analyze the differences between the DIS and TC groups when wearing DIMS.

P3: Independent t-tests were used to analyze the differences between the DIMS and SVL groups when viewing TC.

P4: Independent t-tests were used to analyze the differences between the DIMS and SVL groups when viewing DIS.

P5: Independent t-tests were used to analyze the differences between the DIMS + DIS and SVL + TC groups.

*Significant difference.

## Discussion

Growing evidence shows that the peripheral retina, unlike the central retina, is important for the development of central refractive development (19, 20). As the choroid may play a crucial role in altering its thickness and transducing retinal signals (21, 22), it is important to understand how the choroid responds spatially to different stimuli for further investigation of myopia control. In this prospective, randomized, crossover, and pilot clinical trial, we used high-resolution SS-OCT imaging to evaluate the regional changes in the ChT of children’s eyes induced by short-term DIS usage and near work when wearing DIMS or SVL. We found that greater choroidal thickening changes occur within the temporal and inferior regions in children wearing SVL in the DIS group compared to the TC group. For children using TC, the DIMS group showed thicker choroids in several regions compared to those wearing SVL as a control. The combination of DIMS and DIS exhibited greater ChT thickening in the inferior region versus the SVL and TC groups. Additionally, we observed a transient increase in SER when wearing SVL, similar to the myopia shifts caused by near work in studies from India and China (8, 9). There were no SER changes observed in either the DIMS or DIS groups, indicating that DIMS or DIS devices may partially counteract the transient myopia induced by near work.

As the time that children spent focusing on short-vision tasks and digital devices surged, optical devices that extend viewing distances have become a research focus in recent years. Lin and Yeh et al. (23, 24) proposed a double-mirror system that extends the image from 0.4 m to 2.28 m by using one concave mirror and one convex mirror, resulting in a reduction in the accommodative response compared with traditional near work. Ma et al. (25) used a long-distance optical image workbench (LOIW) to generate upright magnified virtual images of the text at a distance of 3 m, which can replace traditional near-distance reading while also thickening the choroid. In our study, the DIS extended the viewing distance to 5 meters and enlarged the images on the LCD display using two/three optical elements, making switching between images and videos more convenient than with the above devices.

The study compared the effects of short-term DIS use and near work on choroidal regional variations in young children. Whether near work contributes to the ChT response has been controversial. Liu et al. found that 20 min of near work significantly reduced subfoveal ChT in adults (26). While one study indicated that a duration of 40 min of near work decreased macular ChT in nine regions, the differences were not statistically significant (27). In addition, the relationship between LOIW and accommodation has been reported, and children viewed through LOIW exhibited reduced accommodative lag and microfluctuations, suggesting that a virtual optical device provided an appropriate accommodative relaxation (25). The asymmetry in the choroidal modulation has been reported in a study of accommodation stimuli (28), which showed significant choroidal thinning in the temporal and inferotemporal regions, and the underlying mechanism may be related to the distribution of non-vascular smooth muscle cells predominantly on the temporal side. Similarly, our study found that the DIS group exhibited choroidal thickening in the temporal and inferior choroid compared to the TC group when wearing SVL, and that the structural and functional mechanisms require further elucidation.

There is evidence that the human eye can respond to retinal image defocus by changing its axial length within minutes (29), and that thickening of the choroid and a reduction in axial length were observed after 60 min of myopic defocus (13). The finding of short-term choroidal thickening associated with optical devices and lenses may have potential implications for the development of myopia. In eyes that use large amounts of DIS, the choroid will be thickened more frequently and for a longer period of time, which may predispose the eye to longer-term eye changes in growth. Given that DIS provided an extended viewing distance and induced regional choroidal thickening, its use may exert anti-myopic effects.

Recent studies have demonstrated that myopic defocus has long-term effects on the choroid (30, 31). However, there have been conflicting results regarding choroid effects when exposed to short-term myopic defocus. For example, after imposing lens defocus, several studies found increased ChT when viewing at a distance (32–34), while other studies reported no significant thickening (35–37), which may be due to a wide variation in experimental protocols and numerous variables. Using the described protocol, our study simulated long-distance viewing with DIS and found no difference between the DIMS and SVL groups. Interestingly, if participants viewed a TC at a near distance, we observed significant changes in choroidal thickness in the central, inferior, nasal, and temporal regions in the DIMS group. In terms of regional changes in ChT in response to myopic defocus, animal studies have shown that the inferior-nasal choroid of the chick is more responsive than the other regions of the choroid (38). In adults, myopia control contact lenses with +1.50 D addition increased ChT in sub-foveal, temporal, and nasal regions compared to single-vision contact lenses (39). Given the limited research on regional changes in ChT during near work, this topic merits further exploration.

We observed that the combination of DIMS and DIS showed greater ChT changes in the inferior region than in the SVL and TC groups. Based on the above results, DIS and DIMS alone also induced greater changes in the inferior region. Collectively, these results suggest that the inferior region of the choroid is sensitive to both near work and myopic defocus.

The strength of this study was the crossover design, which allowed all participants to experience both devices and lens types, enabling comparisons both across and within groups. However, the current study also has some limitations. This was a pilot study; the sample size was relatively limited. Additionally, we only analyzed the choroidal changes observed after 90 min during the experimental session. The choroid is a dynamic structure whose thickness is modulated by numerous physiologic stimuli and visual cues, but such transient thickening does not necessarily reflect sustained structural alterations in the sclera or long-term axial elongation control. To obtain stronger evidence for the new reading mode that DIS enables, larger samples and longitudinal studies of myopia control in daily habitual viewing patterns are warranted.

In summary, this study demonstrated significant regional thickening of the choroid in response to the DIS device or DIMS lenses in school-aged children. This study provides new insights into the effects of optical devices that extend viewing distance on myopia control.

## Data Availability

The original contributions presented in the study are included in the article/Supplementary material, further inquiries can be directed to the corresponding author.

## References

[1] Holden BA Fricke TR Wilson DA Jong M Naidoo KS Sankaridurg P . Global prevalence of myopia and high myopia and temporal trends from 2000 through 2050. Ophthalmology. (2016) 123:1036–42. doi: 10.1016/j.ophtha.2016.01.006, 26875007

[2] Li R Zhang K Li SM Zhang Y Tian J Lu Z . Implementing a digital comprehensive myopia prevention and control strategy for children and adolescents in China: a cost-effectiveness analysis. Lancet Reg Health West Pac. (2023) 38:100837. doi: 10.1016/j.lanwpc.2023.100837, 37520278 PMC10372367

[3] Alvarez-Peregrina C Sanchez-Tena MA Martinez-Perez C Villa-Collar C. The relationship between screen and outdoor time with rates of myopia in Spanish children. Front Public Health. (2020) 8:560378. doi: 10.3389/fpubh.2020.560378, 33178659 PMC7592393

[4] Tsai TH Liu YL Ma IH Su CC Lin CW Lin LLK . Evolution of the prevalence of myopia among Taiwanese schoolchildren: a review of survey data from 1983 through 2017. Ophthalmology. (2021) 128:290–301. doi: 10.1016/j.ophtha.2020.07.017, 32679159

[5] Dutheil F Oueslati T Delamarre L Castanon J Maurin C Chiambaretta F . Myopia and near work: a systematic review and Meta-analysis. Int J Environ Res Public Health. (2023) 20:875. doi: 10.3390/ijerph20010875, 36613196 PMC9820324

[6] Gajjar S Ostrin LA. A systematic review of near work and myopia: measurement, relationships, mechanisms and clinical corollaries. Acta Ophthalmol. (2022) 100:376–87. doi: 10.1111/aos.15043, 34622560

[7] Huang HM Chang DS Wu PC. The association between near work activities and myopia in children-a systematic review and meta-analysis. PLoS One. (2015) 10:e0140419. doi: 10.1371/journal.pone.0140419, 26485393 PMC4618477

[8] Zhen Y Zhang W Shen J Cheng DW Shen WR Wang NL. The clinical value of using a distant-image screen for reading and learning. Zhonghua Yan Ke Za Zhi. (2022) 58:1045–50. doi: 10.3760/cma.j.cn112142-20220106-00004, 36480886

[9] Lam CSY Tang WC Tse DY Lee RPK Chun RKM Hasegawa K . Defocus incorporated multiple segments (DIMS) spectacle lenses slow myopia progression: a 2-year randomised clinical trial. Br J Ophthalmol. (2020) 104:363–8. doi: 10.1136/bjophthalmol-2018-313739, 31142465 PMC7041503

[10] Jin P Zou H Xu X Chang TC Zhu J Deng J . Longitudinal changes in choroidal and retinal thicknesses in children with myopic shift. Retina. (2019) 39:1091–9. doi: 10.1097/IAE.0000000000002090, 29517579 PMC6553975

[11] Chiang ST Phillips JR Backhouse S. Effect of retinal image defocus on the thickness of the human choroid. Ophthalmic Physiol Opt. (2015) 35:405–13. doi: 10.1111/opo.12218, 26010292

[12] Wang D Chun RK Liu M Lee RP Sun Y Zhang T . Optical defocus rapidly changes choroidal thickness in schoolchildren. PLoS One. (2016) 11:e0161535. doi: 10.1371/journal.pone.0161535, 27537606 PMC4990278

[13] Read SA Collins MJ Sander BP. Human optical axial length and defocus. Invest Ophthalmol Vis Sci. (2010) 51:6262–9. doi: 10.1167/iovs.10-5457, 20592235

[14] Nava DR Antony B Zhang LI Abramoff MD Wildsoet CF. Novel method using 3-dimensional segmentation in spectral domain-optical coherence tomography imaging in the chick reveals defocus-induced regional and time-sensitive asymmetries in the choroidal thickness. Vis Neurosci. (2016) 33:E010. doi: 10.1017/S0952523816000067, 27485367

[15] Chiang ST Phillips JR. Effect of atropine eye drops on choroidal thinning induced by hyperopic retinal defocus. J Ophthalmol. (2018) 2018:8528315. doi: 10.1155/2018/8528315, 29576882 PMC5821954

[16] Chen Z Xue F Zhou J Qu X Zhou X. Effects of orthokeratology on choroidal thickness and axial length. Optometry Vision Sci. (2016) 93:1064–71. doi: 10.1097/OPX.0000000000000894, 27273270

[17] Alanazi M Caroline P Alshamrani A Liu M. Impact of multifocal gas-permeable lens designs on short-term choroidal response, axial length, and retinal defocus profile. Int J Ophthalmol. (2024) 17:247–56. doi: 10.18240/ijo.2024.02.04, 38371246 PMC10827623

[18] Chun RKM Zhang H Liu Z Tse DYY Zhou Y Lam CSY . Defocus incorporated multiple segments (DIMS) spectacle lenses increase the choroidal thickness: a two-year randomized clinical trial. Eye Vis (Lond). (2023) 10:39. doi: 10.1186/s40662-023-00356-z, 37715201 PMC10502972

[19] Troilo D Smith EL 3rd Nickla DL Ashby R Tkatchenko AV Ostrin LA . IMI - report on experimental models of emmetropization and myopia. Invest Ophthalmol Vis Sci. (2019) 60:M31–88. doi: 10.1167/iovs.18-25967, 30817827 PMC6738517

[20] Benavente-Perez A Nour A Troilo D. Axial eye growth and refractive error development can be modified by exposing the peripheral retina to relative myopic or hyperopic defocus. Invest Ophthalmol Vis Sci. (2014) 55:6765–73. doi: 10.1167/iovs.14-14524, 25190657 PMC4209715

[21] Nickla DL Wallman J. The multifunctional choroid. Prog Retin Eye Res. (2010) 29:144–68. doi: 10.1016/j.preteyeres.2009.12.002, 20044062 PMC2913695

[22] Schaeffel F Swiatczak B. Mechanisms of emmetropization and what might go wrong in myopia. Vis Res. (2024) 220:108402. doi: 10.1016/j.visres.2024.108402, 38705024

[23] Lin SY Su HR Lo CC Yeh SM Lee CH Wu R . Effects of extended viewing distance on accommodative response and pupil size of myopic adults by using a double-Mirror system. Int J Environ Res Public Health. (2022) 19:2942. doi: 10.3390/ijerph19052942, 35270634 PMC8910498

[24] Yeh SM Lo CC Lee CH Chen YJ Lin FC Huang SY. Reduction in accommodative response of schoolchildren by a double-Mirror system. Int J Environ Res Public Health. (2021) 18:9951. doi: 10.3390/ijerph18199951, 34639251 PMC8508213

[25] Ma L Li X Hu J Li Y Wang S Wang K . Influence of a long-distance optical imaging workbench on accommodation and choroidal response in myopic children. Clin Exp Optom. (2024) 107:420–7. doi: 10.1080/08164622.2023.2228810, 37406457

[26] Liu M Wang Y Li H Zhao Y Ma M Xu S . Differences in choroidal responses to near work between myopic children and young adults. Eye Vis (Lond). (2024) 11:12. doi: 10.1186/s40662-024-00382-5, 38561862 PMC10986059

[27] Liang X Wei S Zhao S Li SM An W Sun Y . Investigation of choroidal blood flow and thickness changes induced by near work in young adults. Curr Eye Res. (2023) 48:939–48. doi: 10.1080/02713683.2023.2222234, 37303164

[28] Woodman-Pieterse EC Read SA Collins MJ Alonso-Caneiro D. Regional changes in choroidal thickness associated with accommodation. Invest Ophthalmol Vis Sci. (2015) 56:6414–22. doi: 10.1167/iovs.15-17102, 26444722

[29] Delshad S Collins MJ Read SA Vincent SJ. The time course of the onset and recovery of axial length changes in response to imposed defocus. Sci Rep. (2020) 10:8322. doi: 10.1038/s41598-020-65151-5, 32433541 PMC7239843

[30] Alvarez-Peregrina C Sanchez-Tena MA Martinez-Perez C Villa-Collar C Clinical Evaluation of MyoCare in Europe -theCSG Ohlendorf A. Clinical evaluation of MyoCare in Europe (CEME): study protocol for a prospective, multicenter, randomized, double-blinded, and controlled clinical trial. Trials. (2023) 24:674. doi: 10.1186/s13063-023-07696-0 PMC10580514 37848908

[31] Li X Hu J Peng Z Chen S Sun L Wang K . Association between choriocapillaris perfusion and axial elongation in children using defocus incorporated multiple segments (DIMS) spectacle lenses. Eye (Lond). (2023) 37:3847–53. doi: 10.1038/s41433-023-02629-2, 37369765 PMC10697950

[32] Hoseini-Yazdi H Vincent SJ Read SA Collins MJ. Astigmatic defocus leads to short-term changes in human choroidal thickness. Invest Ophthalmol Vis Sci. (2020) 61:48. doi: 10.1167/iovs.61.8.48, 32729913 PMC7425733

[33] Hoseini-Yazdi H Vincent SJ Collins MJ Read SA. Regional alterations in human choroidal thickness in response to short-term monocular hemifield myopic defocus. Ophthalmic Physiol Opt. (2019) 39:172–82. doi: 10.1111/opo.12609, 30950105

[34] Sander BP Collins MJ Read SA. The interaction between homatropine and optical blur on choroidal thickness. Ophthalmic Physiol Opt. (2018) 38:257–65. doi: 10.1111/opo.12450, 29691923

[35] Kubota R Joshi NR Samandarova I Oliva M Selenow A Gupta A . Effect of short-term peripheral myopic defocus on ocular biometrics using Fresnel "press-on" lenses in humans. Sci Rep. (2021) 11:22690. doi: 10.1038/s41598-021-02043-2, 34811408 PMC8608875

[36] Ostrin LA Sah RP Queener HM Patel NB Tran R Shukla D . Short-term myopic defocus and choroidal thickness in children and adults. Invest Ophthalmol Vis Sci. (2024) 65:22. doi: 10.1167/iovs.65.4.22, 38597724 PMC11008753

[37] Swiatczak B Schaeffel F. Emmetropic, but not myopic human eyes distinguish positive defocus from calculated blur. Invest Ophthalmol Vis Sci. (2021) 62:14. doi: 10.1167/iovs.62.3.14, 33687476 PMC7960797

[38] Huang Y Wang Y Shen Y Chen Z Peng X Zhang L . Defocus-induced spatial changes in choroidal thickness of chicks observed by wide-field swept-source OCT. Exp Eye Res. (2023) 233:109564. doi: 10.1016/j.exer.2023.109564, 37419380

[39] Amorim-de-Sousa A Paune J Silva-Leite S Fernandes P Gozalez-Meijome JM Queiros A. Changes in choroidal thickness and retinal activity with a myopia control contact Lens. J Clin Med. (2023) 12:12. doi: 10.3390/jcm12113618, 37297813 PMC10253444

