# Supplementary material for: Short-term effects of distant-image screen and myopic defocus on choroidal thickness in children: a randomized pilot clinical trial
Source: Front Public Health. 2026 Sep 4;14:1884932. doi: 10.3389/fpubh.2026.1884932 (PMC13587909; doi:10.3389/fpubh.2026.1884932)
Supplement: Supplementary file 1 [file Data_Sheet_1.pdf]

SAS Randomized Table

The random number is preferentially assigned according to the order in which the subject signs the informed consent, that is, the entry order: if the first participant signs the informed consent, corresponding to the number 1 in the entry order below, is assigned to the group of SVL+DIS.

**Obs**	**block**	**length**	**Entry order**	**Result**
**1**	9	2	1	SVL+DIS
**2**	9	4	2	DIMS+DIS
**3**	9	3	3	DIMS+TC
**4**	9	1	4	SVL+TC
**5**	10	3	5	DIMS+TC
**6**	10	2	6	SVL+DIS
**7**	10	1	7	SVL+TC
**8**	10	4	8	DIMS+DIS
**9**	5	3	9	DIMS+TC
**10**	5	1	10	SVL+TC
**11**	5	2	11	SVL+DIS
**12**	5	4	12	DIMS+DIS
**13**	6	4	13	DIMS+DIS
**14**	6	3	14	DIMS+TC
**15**	6	2	15	SVL+DIS
**16**	6	1	16	SVL+TC
**17**	2	1	17	SVL+TC
**18**	2	3	18	DIMS+TC
**19**	2	4	19	DIMS+DIS
**20**	2	2	20	SVL+DIS
**21**	3	4	21	DIMS+DIS
**22**	3	1	22	SVL+TC
**23**	3	3	23	DIMS+TC
**24**	3	2	24	SVL+DIS
**25**	8	2	25	SVL+DIS
**26**	8	1	26	SVL+TC
**27**	8	4	27	DIMS+DIS
**28**	8	3	28	DIMS+TC
**29**	1	2	29	SVL+DIS
**30**	1	3	30	DIMS+TC
**31**	1	4	31	DIMS+DIS
**32**	1	1	32	SVL+TC

**33**	4	4	33	DIMS+DIS
**34**	4	3	34	DIMS+TC
**35**	4	2	35	SVL+DIS
**36**	4	1	36	SVL+TC
**37**	7	1	37	SVL+TC
**38**	7	4	38	DIMS+DIS
**39**	7	3	39	DIMS+TC
**40**	7	2	40	SVL+DIS

SVL: single-vision lenses; DIMS: defocus incorporated multiple segments lenses; TC: tablet computer; DIS: distance-image screen.
