# Supplementary material for: Short-term effects of distant-image screen and myopic defocus on choroidal thickness in children: a randomized pilot clinical trial
Source: Front Public Health. 2026 Sep 4;14:1884932. doi: 10.3389/fpubh.2026.1884932 (PMC13587909; doi:10.3389/fpubh.2026.1884932)
Supplement: Supplementary file 2 [file Table_1.pdf]

Table Baseline choroidal thickness at different stages in two groups (mean±SE)

Regions	SVL			DIMS		
	Baseline at stage I	Baseline at stage II	*P*	Baseline at stage I	Baseline at stage II	*P*
**C (μm)**	283.74±58.84	283.74±56.99	1.000	260.45±48.80	263.25±39.53	0.736
**S3 (μm)**	270.60±60.40	281.74±51.00	0.318	262.05±52.11	265.65±49.54	0.599
**I3 (μm)**	276.55±73.17	276.20±69.98	0.907	267.10±48.99	267.65±41.68	0.949
**N3 (μm)**	240.95±67.36	243.60±65.55	0.282	231.10±51.36	233.30±44.56	0.801
**T3 (μm)**	286.10±58.62	284.90±58.87	0.586	286.10±58.62	272.30±42.15	0.815
**S6 (μm)**	275.60±52.88	272.05±43.27	0.178	264.35±53.76	266.35±51.55	0.763
**I6 (μm)**	270.10±77.50	273.35±66.52	0.518	263.80±44.64	267.55±42.19	0.638
**N6 (μm)**	196.60±52.45	197.85±52.19	0.570	184.50±47.50	189.05±44.89	0.630
**T6 (μm)**	285.90±46.82	286.15±48.62	0.891	275.15±50.54	276.85±48.35	0.805

C, 1×1 mm circular region; S3, I3, N3 and T3, the superior, inferior, nasal and temporal regions measured at 1-3 mm; S6, I6, N6 and T6, the superior, inferior, nasal and temporal regions measured at 3-6 mm;

SVL: single-vision lenses; DIMS: defocus incorporated multiple segments lenses; TC: tablet computer; DIS: distance-image screen.
